# Mastering Immunity: Antibody Feedback as a Driver of Germinal Center Fate and Vaccine Responses

**DOI:** 10.1002/eji.70108

**Published:** 2025-12-19

**Authors:** Shuang Liu, Yang Zhang, Kai‐Michael Toellner

**Affiliations:** ^1^ Emergency Department State Key Laboratory of Complex Severe and Rare Disease Peking Union Medical College Hospital Chinese Academy of Medical Science and Peking Union Medical College Beijing China; ^2^ Immunology Program Babraham Institute Cambridge UK

**Keywords:** affinity maturation, antibody feedback, epitope spread, germinal center, vaccination

## Abstract

Antibody feedback in germinal center (GC) responses plays a key role in shaping the affinity, specificity, and longevity of humoral immunity. Beyond neutralizing pathogens, antibodies influence B cell selection by modulating antigen availability and masking dominant epitopes, thereby reshaping the competitive landscape for T follicular helper (Tfh) cell support. This review outlines the current understanding of how antibody feedback governs the selection stringency and clonal evolution of GC B cells, facilitates and promotes the emergence of epitope spread, and contributes to GC shutdown. We also examine how it supports the development of broadly neutralizing antibodies. Finally, we discuss how these insights are informing next‐generation vaccine strategies—including immunogen design, prime‐boost regimens, and adjuvant optimization—to guide affinity maturation toward specific epitopes and overcome feedback‐driven constraints. Understanding antibody feedback not only reveals fundamental principles of adaptive immunity but also offers new avenues for rational vaccine design and therapeutic immune modulation.

## Graphical abstract

Antibody feedback dynamically shapes the selective landscape for GC B‐cells, acting as a driving force of Darwinian‐like affinity maturation of B‐cell receptors (A). It also balances epitope usage, leading to equal competition among B cell clones specific for different epitopes and allowing the emergence of clones specific for new epitopes (B).

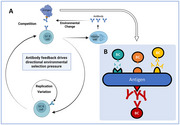

**Abbreviations**
ASC: antibody‐secreting cells
BCR: B cell receptor
BnAb: broadly neutralizing antibody
FDCs: follicular dendritic cells
GC: germinal center
MHCII: major histocompatibility complex II
Tfh: T follicular helper

## 1 Introduction

Antibodies, beyond functioning as terminal effectors of humoral immunity, can actively regulate B cell responses by altering antigen and specific epitope availability and modulating the selection pressure for affinity maturation [1, 2, 3]. Whether B cells bind antigen is not only dependent on their specificity and affinity, but also on whether they can compete with epitope‐specific antibodies that may be present from a previous response [2, 4, 5]. The concept dates back more than 100 years, when it was found that passively transferred antibodies could modulate immune protection in vaccination [6]. Since then, studies have confirmed that antibodies can exert feedback regulation on the very immune responses they originate from, amplifying or suppressing antibody production by several orders of magnitude depending on context [3, 7, 8, 9, 10, 11, 12].

**FIGURE 1:**
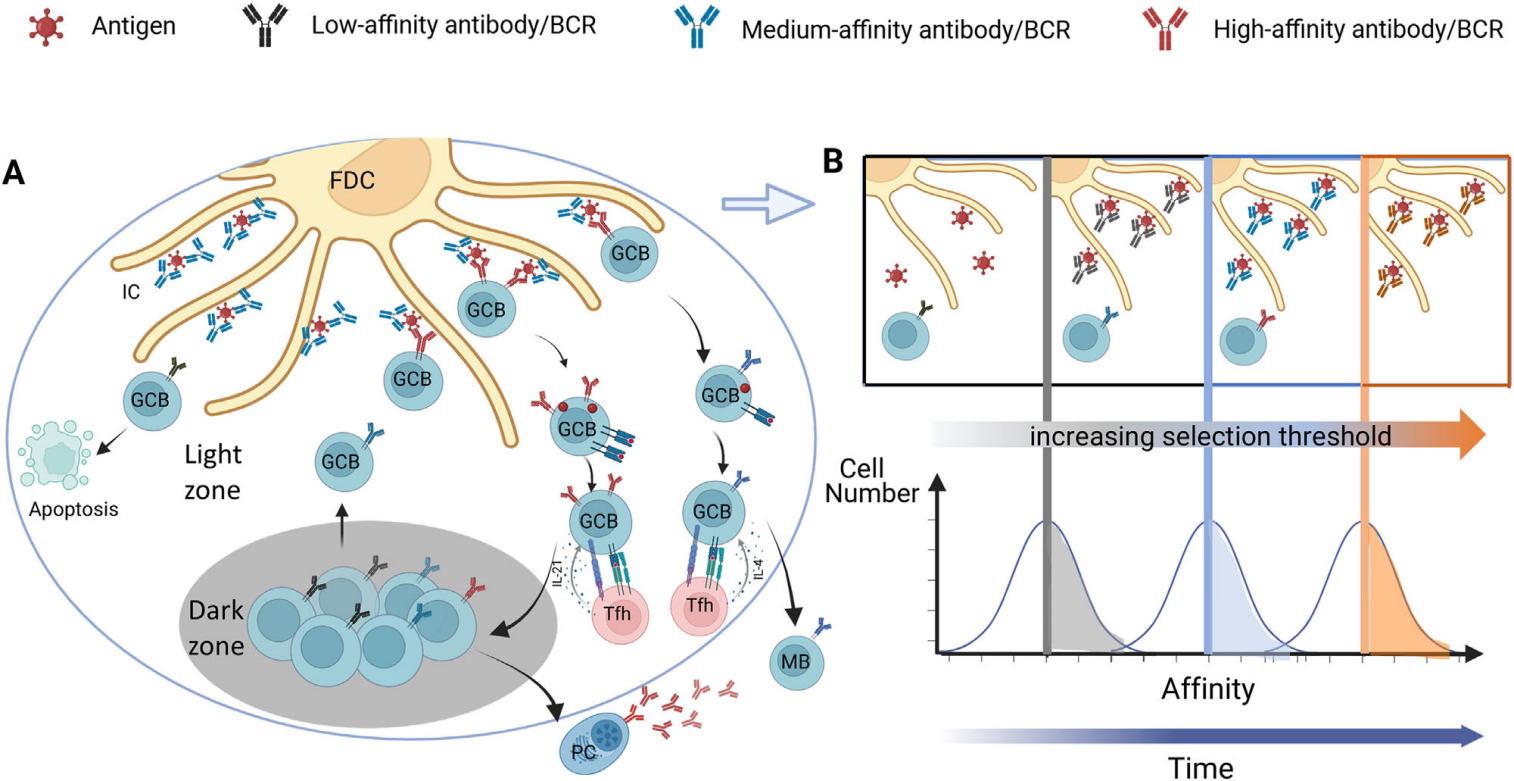
(A) Antibody‐mediated affinity maturation. Antigens are deposited on FDCs in the form of immune complexes (ICs). GC B cells acquire antigen by competing with epitope‐specific antibodies in ICs that reduce the effective free antigen concentration. B cells failing to compete may die by apoptosis, whereas B cells acquiring antigen receive Tfh cell help that is dependent on the amount of MHCII‐peptide presented to Tfh cells. High‐affinity cells will recycle into the dark zone and ultimately produce a higher‐affinity antibody that may reenter the GC. Some GC B cells differentiate into plasma cells (PCs), which secrete antibodies, or memory B cells (MBs), which provide long‐term immunity. (B) Directional evolution in the GC. Initial low‐affinity antibodies are progressively replaced by higher‐affinity antibodies generated by GC‐derived plasma cells. This will reduce effective antigen concentration and lead to a gradually rising selection threshold. This schematic figure has been simplified to show the principle of gradual antibody replacement: many effects of antibodies described in this review were tested using IgM and not monovalent IgG. Complement and its role in binding of immune complexes to FDC or activating B cells are not shown. Similarly, the interactions of the antibodies with individual epitopes are only shown symbolically.

Following antigen encounter, B cells differentiate via the extrafollicular pathway—resulting in rapid plasma cell generation but typically limited affinity maturation [13, 14]—or migrate into follicles, where they undergo iterative cycles of affinity maturation within the GC [15]. The GC involves clonal expansion and somatic hypermutation in the dark zone, followed by antigen‐driven and Tfh cell‐mediated B cell selection in the light zone (Figure 1). Survival and fate decisions depend on two key interactions: acquisition of antigen from follicular dendritic cells (FDCs) and stimulation by Tfh cells. GC B cells are intrinsically programmed for apoptosis unless rescued by B cell receptor (BCR) engagement and sufficient Tfh‐derived signals, which guide their recycling within the GC, differentiation into plasma cells, or transition into memory B cells [16, 17].

Interestingly, antibodies—including those secreted by GC‐derived plasma cells—can re‐enter this cycle and influence selection outcomes by regulating epitope accessibility [18]. Early GC‐derived antibodies establish a dynamic feedback loop that regulates antigen availability for subsequent B cell selection [6, 9, 12]. By binding antigen, they influence its retention and display on FDCs, reshaping the competitive landscape for B cell selection and modulating Tfh cell help. In recall GCs, pre‐existing antibodies further determine the balance between recruiting naïve versus memory B cells, thereby steering the trajectory of affinity maturation [5, 10, 12]. Through these mechanisms, antibody feedback fine‐tunes B cell selection and preserves immune homeostasis, providing a conceptual basis for vaccine strategies that aim to direct GC evolution toward durable and broad protection [11, 12].

Despite the growing recognition of its importance, the mechanisms of antibody feedback in the GC remain poorly understood. The key unresolved questions include: How do thresholds set by antibody affinity dynamically reshape antigen accessibility during GC maturation? How does antibody feedback modulate the availability of individual epitopes? How does this modulate Tfh‐mediated B cell selection? How does it influence the recruitment of naïve versus memory B cells during the onset of recall responses? Resolving these mechanisms will advance our understanding of GC regulation and guide vaccine strategies that exploit antibody feedback to achieve durable and broad protection.

## 2 Impact of Antibodies on GCs

### 2.1 Antibody Feedback Driving Affinity Maturation in the GC

B‐cell differentiation in GCs is a biological analogue to Darwinian evolution of species at a cellular level (Figure 1). It is driven by the same three mechanisms: iterations of cellular reproduction, accompanied by variation through mutation, and natural selection [19]. Antibody feedback has been implicated in shaping the selection environment, driving the directional evolution of affinity maturation in the GC. Antigen‐specific B cells clonally expand and hypermutate their antibody variable region genes in the GC dark zone through immunoglobulin gene hypermutation [16]. Hypermutation generates random changes in BCR affinity, making a subsequent selection step a necessary. Selection happens on FDCs in the GC light zone through antigens presented in the form of large deposits of immune complexes on the FDC network [20]. This provides an opportunity for the interaction of mutated BCRs with their specific epitopes. GC initiation as well as maintenance have been shown to depend on the presence of antigen immune complexes [21]. Early antigen‐specific antibody, secreted during the early extrafollicular plasma cell response [22, 23], may facilitate antigen deposition on the FDC network. Antibody in these immune complexes not only facilitates deposition of antigen on FDCs, but they also provide a barrier for antigen access. As the affinity of the antibodies increases over time, this will generate a rising selection threshold leading to directional evolution toward higher affinity.

B‐cell selection is dependent on the quality of the interaction with Tfh cells. There is convincing evidence that only B cells presenting high amounts of cognate MHCII—peptide complexes will receive adequate signals from Tfh cells (e.g., CD40L and IL‐21) that drive re‐entry into the dark zone [17, 24, 25], leading to further clonal expansion and hypermutation. While Tfh signals are critical, the evolutionary trajectory of B cells is determined by the increase in affinity of their BCR for antigen presented on FDCs. B cells can sense BCR affinity when interacting with antigen at concentration in the nM to pM range, which can trigger affinity‐dependent antigen uptake, presentation, and subsequent Tfh cell activation [26].

If such small concentrations of antigen determine affinity‐dependent selection, how can the excessively high antigen concentrations on the surface of FDCs still create an affinity‐selecting environment? An explanation may be that epitopes in these immune complexes are not freely accessible but are shielded by epitope‐specific antibody. Antibodies in the GC are initially of low affinity but are gradually replaced by affinity‐matured variants secreted by plasma cells emerging from ongoing GC activity [27]. This progressive replacement would reduce the effective concentration of free antigen to sub‐nM levels, generating conditions that favor affinity‐dependent B cell selection, and leading to a dynamic increase in selection pressure that rises as the GC matures (Figure 1B). Effects of antibody feedback on affinity maturation have been tested by passively transferring exogenous antibody after active immunization, which confirmed such effects [18, 28]. Such a gradual adjustment of selection pressure mirrors principles of gradual adaptation in Darwinian evolution [29], but operates through a dynamic immunological feedback rather than through selection pressures generated by a changing natural environment.

### 2.2 Effects of Antibody Feedback via Immune Complexes on FDCs

In addition to its effects on driving affinity maturation, antibodies can reshape the antigenic landscape within GCs [30]. Epitope masking refers to the process by which antibody binding to specific epitopes blocks their accessibility to BCRs, thereby altering which epitopes remain available for recognition. Epitope masking can leave subdominant or cryptic epitopes exposed, enabling rare or low‐affinity B cell clones specific for such epitopes to participate in the GC reaction [27, 28, 31, 32]. This shift in antigen availability diversifies the B cell repertoire and broadens immune protection. A 3D structural model of antibody‐antigen binding affinity embedded in GC computer simulations supports that epitope masking modulates GC clonal dynamics [33],

Antibody‐mediated epitope masking can reshape the competitive dynamics within GCs by determining which B cells gain sufficient access to downstream Tfh cell help. Only B cells that capture sufficient antigen and present peptide can effectively engage Tfh cells, receive co‐stimulatory signals, and undergo affinity maturation [4, 24, 25, 34, 35, 36, 37], while others undergo apoptosis or premature differentiation into memory B cells [34, 38, 39]. Although high‐affinity B cells dominate by presenting more peptide–MHCII complexes, masking of dominant epitopes allows subdominant clones to acquire more antigen, secure Tfh support, and diversify the emerging antibody repertoire [27, 31].

**FIGURE 2:**
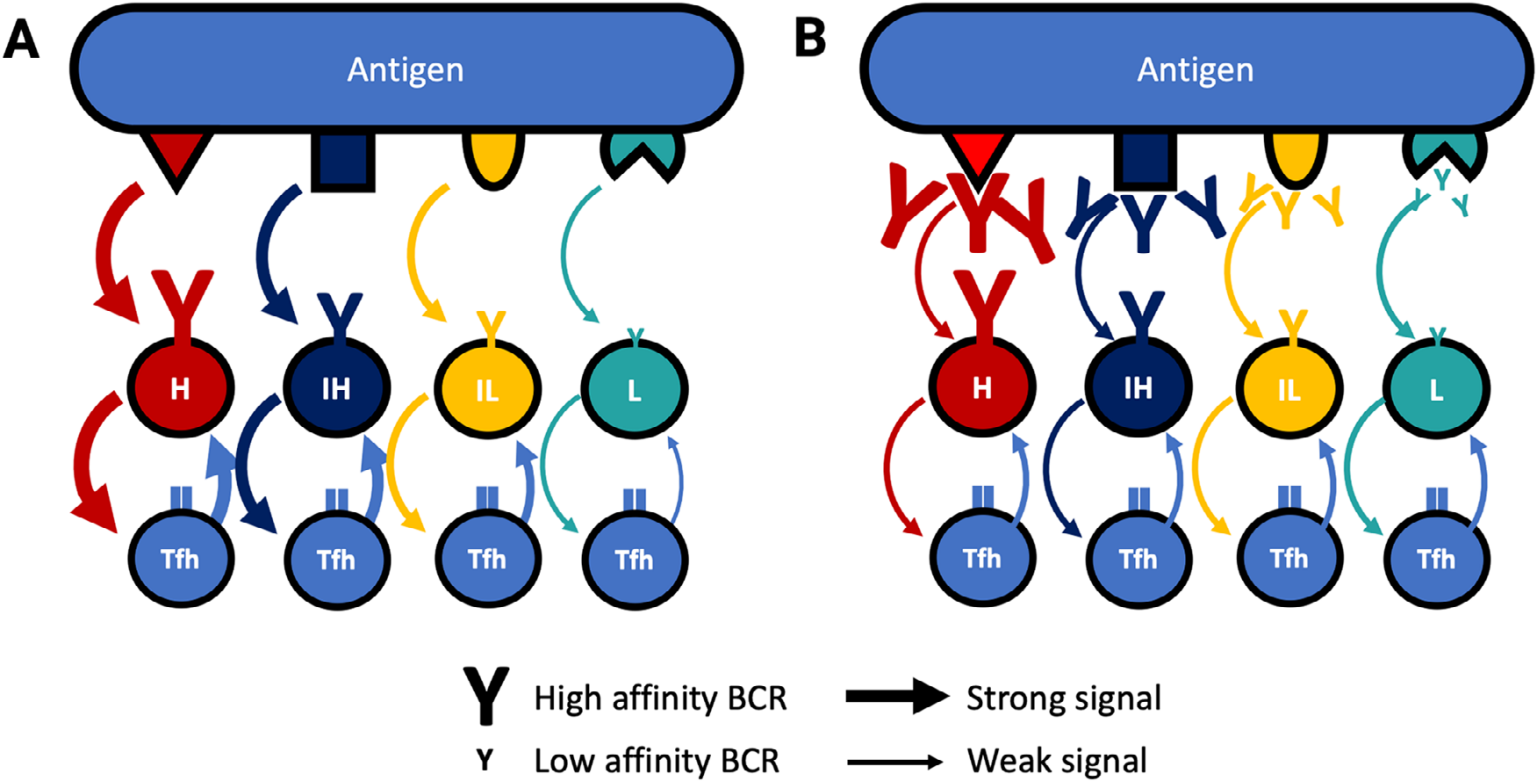
Antibody feedback generates different handicaps for B‐cell clones competing for the same Tfh cells. (A) In a situation where different affinity B cell clones compete for different epitopes on the same antigen, higher‐affinity clones should have a competitive advantage. This does not fit experimental observations [41]. (B) As antibody feedback causes competition of B cells with their own outputs (affinity‐matured antibodies), higher‐affinity B cells will encounter stricter selection than clones binding epitopes that are still free or covered by low‐affinity antibody. This will allow low‐affinity B cells to compete with equivalent efficiency for help from Tfh cells. H: High, I: intermediate, L: low‐affinity B cell, Tfh: Tfh cell. Colour codes for antigen specificity. The sizes of antibodies and arrows code for affinity and strength of interaction. Note that all Tfh cells (blue) have identical specificity for the same antigen (blue). *Source*: Modified from the study by Toellner et al. [27].

Early experiments using immunization with complex protein antigens showed a surprisingly large spread of affinities in individual GC B cells specific for the same antigen [40]. This seemed incompatible with the idea of a general selection threshold imposed by antibody feedback. However, later studies that were not blind to epitope specificities showed that selection thresholds for members of the same B cell clone (specific for the same epitope) were far tighter [41]. This shows that there is epitope‐specific, not antigen‐specific, competition between GC B cells. This leads to an attractive model (Figure 2), where antibody feedback ensures that B cells of different affinities, specific for different epitopes, can compete for the same antigen on equal terms, while both compete for survival signals from the same Tfh cells [27].

Apart from restricting epitope accessibility, antibodies augment antigen deposition on FDCs and, in this way, allow more B cells to capture antigen sufficient for subsequent selection by Tfh cells. Antibodies multimerize antigens and increase antigen valency, which is a key determinant of BCR signaling strength and B cell activation [42]. Experimental comparisons of HIV immunogens with differing valencies have shown that higher‐valence antigens can effectively recruit lower‐affinity B cells into the GC reaction [43]. By increasing antigen valency, antibody‐ and complement‐mediated opsonization may promote inclusion of lower‐affinity clones, enhance epitope diversity, and broaden the antibody repertoire [34].

Immune complexes enhance antigen retention on FDCs through complement activation. Complement‐opsonized immune complexes further amplify BCR signaling via the CR2/CD19/CD81 co‐receptor complex, lowering the activation threshold for B cells [44], while engagement of inhibitory FcγRIIB can attenuate B cell activation and promote tolerance [45]. Crosslinking of the BCR with the CR2/CD19/CD81 complex can increase B cell sensitivity to antigen by over 1000‐fold, as shown in C3d‐conjugated antigen models that mimic complement‐opsonized immune complexes [46, 47], and CR2 ligation on B cells significantly boosts responses to various antigens [48, 49]. While CR1/2‐mediated co‐stimulation strongly enhances activation of naïve B cells, its role in GC B cells remains unclear, as CR2 transcript expression is downregulated in these cells [2], which may ensure that antigen capture is primarily determined by BCR affinity rather than complement opsonization. These complementary mechanisms of antigen augmentation and epitope restriction illustrate how antibody feedback may finely tune downstream B cell–Tfh cell interactions to balance affinity selection with repertoire breadth.

### 2.3 Antibody Feedback Mediated Modulation of recall GCs

Epitope masking by natural antibodies during the primary B cell response can limit the availability of cognate antigen, thereby suppressing the activation of naïve B cells and restricting their entry into GCs [5, 27, 31, 34, 50, 51, 52]. Memory B cells tend to differentiate into plasma cells during recall responses, while GCs are dominated by naïve B cells targeting new epitopes [53]. This can be caused by antibodies from the primary response exerting profound effects on recall GC dynamics [5], modulating the recruitment of naïve and memory B cells [32]. Consequently, recall GCs become biased toward naïve or memory B cells that recognize new or drifted epitopes, shaping the antibody repertoire toward previously unrecognized specificities [27, 31]. This creates selective niches that promote the expansion of clones with atypical specificities—a mechanism particularly valuable for vaccine strategies aimed at eliciting broadly neutralizing antibodies [5, 54].

Antibody feedback can restrict memory B cell participation in GCs by blocking access of memory clones to their target epitopes, limiting their ability to acquire antigen and effectively compete for Tfh help. This may lead to their exclusion from the GC reaction, thereby shifting the GC composition toward naive or less‐experienced B cell populations, particularly under conditions of antigenic variation or drift [4, 27, 31, 51, 55].

These principles also apply to memory B cells from responses to different antigens. In an example of SARS‐CoV‐2 mRNA vaccination of COVID‐naïve individuals, the early burst of antibody‐secreting cells (ASC) originated largely from preexisting, cross‐reactive memory B cells—likely primed by past infections with seasonal human coronaviruses. These cells rapidly generated ASCs without re‐entering GCs. Naïve B cells engaged in GC reactions, underwent affinity maturation, and produced new, high‐affinity SARS‐CoV‐2–specific memory and plasma cells [56].

### 2.4 Antibody‐Driven GC Contraction and Shutdown

As GC responses progress, antibody titers and affinities progressively rise, leading to increased epitope masking by antibodies of increasing affinity. This may lead to diversification in epitope usage, resulting in the expansion of subdominant B cell clones specific for epitopes not yet covered by antibodies. At a certain point, further affinity gains may become too difficult, leaving B cells unable to compete with the rising levels of high‐affinity antibodies. High‐affinity antibodies can have dissociation rates so low that it may take B cells days to access sufficient antigen to gain Tfh cell help [27]. The result of reduced availability of free antigen is a reduction in proliferation, increased apoptosis, and ultimately GC contraction—a gradual decline in GC size and activity [18, 27, 57]. This contraction phase, if sustained, culminates in GC shutdown and the complete resolution of the GC reaction.

Antibody‐driven shutdown helps the timely termination of the response, preventing prolonged or dysregulated GC activity that could otherwise result in ineffective antibody production, impaired immune regulation, or potential lymphomagenic transformation [58]. In this way, antibody‐mediated shutdown enforces both efficiency, self‐tolerance, and/or genetic stability. Importantly, antibody‐driven GC contraction is not a uniform or indiscriminate process. Antibodies targeting immunodominant epitopes may selectively suppress B cell clones recognizing those epitopes, while allowing the persistence or expansion of clones directed against nonoverlapping, subdominant, or antigenically drifted epitopes. Both experimental and computational studies have demonstrated that the magnitude and directionality of GC responses can be modulated by exogenous antibodies, with outcomes heavily influenced by the timing, specificity, and concentration of antibody input [18, 28, 31, 59].

This regulatory principle may extend to passive immunization. Therapeutically administered high‐affinity monoclonal antibodies may deposit on FDCs, reshape antigen presentation, and thereby accelerate GC contraction. Similarly, during recall responses, rapid secretion of high‐affinity antibodies by memory‐derived plasma cells with higher affinity can reinforce this feedback loop, promoting faster resolution of GCs specific to those epitopes. While such feedback promotes response efficiency, it may also restrict the breadth of antibody variants recognizing the same epitope, particularly in the absence of new or variant epitopes [30, 60, 61].

It was long unclear whether antibody‐mediated epitope masking or simple antigen clearance was the dominant driver of GC suppression. However, studies using Fc receptor‐deficient models indicate that the suppressive effects of antibody feedback arise primarily from epitope masking rather than antigen elimination per se [9, 45]. These findings underscore the mechanistic importance of epitope‐specific antibody feedback in orchestrating GC contraction and eventual shutdown.

## 3 Translating Antibody Feedback Insights into Next‐Generation Vaccine Design

A growing understanding of antibody feedback may reshape vaccine development. Instead of merely eliciting high antibody titers, next‐generation vaccines aim to guide the quality, specificity, and evolutionary trajectory of humoral responses. Antibody feedback, once seen as a barrier, can be harnessed through interconnected approaches leveraging its regulatory potential within GCs.

### 3.1 Epitope Focusing: Guiding Vaccine‐Induced B Cell Responses

High‐affinity antibodies, particularly IgG isotypes, mask immunodominant regions of pathogens, effectively steering the immune response toward subdominant epitopes. Through this feedback process, antibodies regulate antigen accessibility on FDCs and reshape the competitive landscape for B cell selection, potentially promoting epitope spreading and the generation of broadly protective responses [27, 31]. Given that antibody feedback can markedly lower the concentration of free antigen (to the nM–pM range) during GC evolution, vaccine platforms targeting conserved epitopes must account for this to avoid premature shutdown of low‐affinity B cell clones [27].

Rational immunogen design now deliberately exploits or mitigates this feedback by engineering antigen presentation to maintain selective accessibility of conserved neutralization sites—such as the CD4 binding site on HIV Env or the stem domain of influenza hemagglutinin—while minimizing immune exposure of highly variable, nonprotective epitopes [62, 63]. Moreover, leveraging or inducing early nonneutralizing antibodies in vaccination regimens can suppress off‐target B cell clones through epitope masking, further refining the immune response toward key pathogen vulnerabilities [64].

Finally, epitope‐specific competition illustrates how antibody binding site location dictates outcomes: antibodies recognizing the same or overlapping epitopes as the BCR can block antigen access and suppress B cell activation, disproportionately affecting low‐affinity clones, narrowing GC B cell diversity, and accelerating affinity maturation [52]. In contrast, antibodies targeting nonoverlapping or complementary epitopes avoid such competition, allowing broader B cell recruitment, survival, and sustained affinity maturation [65].

### 3.2 GC Targeting: Sustaining Responses for Improved Vaccine Efficacy

Antibody feedback impacts the quality and longevity of GC responses. Innovative vaccine platforms increasingly aim to sustain GC activity to promote extensive affinity maturation and broadened antibody repertoires. For example, stabilized nanoparticle immunogens improve antigen retention on FDCs, extending GC activity even in the presence of high‐affinity antibody feedback [66]. Additionally, advanced controlled antigen delivery systems—such as slow‐release hydrogels and osmotic pumps—prolong antigen availability within lymphoid tissues, preventing the abrupt antigen–antibody ratio shifts that typically cause premature GC shutdown via antibody feedback [67]. By modulating antibody isotypes and attenuating inhibitory Fc receptor signaling, vaccines can maintain clonal diversity for longer periods, allowing rare and broadly reactive B cell clones to expand and mature [43, 68], once the response to immunodominant epitopes is shut down via antibody feedback. Together, these approaches lead to more durable and broadly protective humoral immunity, critical for effective vaccines against rapidly evolving pathogens.

### 3.3 Heterologous Prime–Boost Strategies: Overcoming Immune Imprinting and Expanding Protection

Original Antigenic Sin—the immune system's preference for recalling memory B cells specific to previously encountered antigenic variants—hinders effective vaccination against mutable viruses [8]. To overcome this, heterologous prime–boost vaccination regimens are designed to leverage antibody feedback mechanisms that suppress dominant memory B cell responses and promote the maturation of naive B cells targeting novel or mutated epitopes [8, 69]. Experimental and computational studies demonstrate that such strategies reshape GC selection dynamics, allowing the immune system to adapt to antigenic drift and broaden protection against evolving viruses such as influenza and SARS‐CoV‐2 [31, 55]. These strategies enable vaccines to “outsmart” immune imprinting, promoting broader, more flexible humoral immune responses critical for long‐term protection.

### 3.4 Novel Adjuvants: Enhancing GC Dynamics and Broadening Immunity

Apart from classical adjuvants relying on innate immune activation through inflammation, the addition of small amounts of monoclonal antibodies to protein or mRNA‐based vaccines can markedly increase their immunogenicity and prolong immune persistence. This “adjuvant” effect works through the formation of immune complexes, which enhance antigen presentation and activation of low‐affinity B cells. This can broaden and enhance B‐cell responses, effectively functioning as a molecular adjuvant to improve vaccine efficacy [65].

## 4 Conclusion

Antibody feedback can mediate positive, negative, or regulatory effects on B cell recruitment, survival, and selection. By enhancing antigen transport and presentation, activating complement, while exposing new epitopes, antibody feedback promotes high‐affinity B cell selection as well as recruitment of naïve clones. Conversely, through epitope masking, Fc gamma receptor–mediated inhibitory signaling, inhibition of memory B cell recruitment, and ultimately GC shutdown, it can restrain excessive diversification. Finally, by restricting effective antigen concentration during affinity maturation in the GC, it acts as a dynamic regulator, imposing directional selection pressure toward higher affinity. A deeper understanding of these feedback mechanisms may provide actionable insights for vaccine design. By carefully controlling antigen dose and timing [70], vaccines may modulate feedback intensity to prolong GC reactions and sustain affinity maturation. Adjusting antigen valency or epitope accessibility may help overcome epitope masking, thereby revealing subdominant or conserved epitopes that drive neutralizing antibody (bnAb) responses broadly [67]. Furthermore, leveraging controlled feedback through sequential or heterologous immunization could selectively steer B cell evolution toward cross‐reactive and long‐lived clones [71]. Such rational manipulation of antibody feedback provides a powerful framework to design next‐generation vaccines with enhanced breadth, durability, and resilience against antigenically variable pathogens.

## Author Contributions

K.T. and Y.Z. conceived the topic and the scope of the review. S.L. and K.T. performed the literature search and synthesized the evidence. S.L. prepared the initial draft. S.L. and K.T. developed the figures. K.T. provided critical revisions, conceptual guidance, and oversight throughout the writing process. All authors contributed to the final editing and approved the submitted version.

## Conflicts of Interest

The authors declare no conflicts of interest.

## Data Availability

Data sharing does not apply to this article as no datasets were generated or analyzed during the current study.
